# Injectable extracellular matrix microparticles for regenerative and biocompatible soft tissue augmentation

**DOI:** 10.17179/excli2026-9490

**Published:** 2026-08-03

**Authors:** Javad Khanali, Yasamin Ostadi, Fatemeh A. Tehrani, Tahereh Tayebi, Roghayeh Tarasi, Younes Yassaghi, Yasaman Nazerian, Maryam Salimi, Feizollah Niazi, Soheyl Bahrami, Hassan Niknejad

**Affiliations:** 1 Department of Pharmacology, School of Medicine, Shahid Beheshti University of Medical Sciences, Tehran, Iran; 2 Department of Biology and Anatomical Sciences, School of Medicine, Shahid Beheshti University of Medical Sciences, Tehran, Iran; 3 Department of Plastic and Reconstructive Surgery, Shahid Beheshti University of Medical Sciences, Tehran, Iran; 4 Ludwig Boltzmann Institute for Experimental and Clinical Traumatology in AUVA Research Center, Vienna, Austria

**Keywords:** amniotic membrane, decellularized extracellular matrix, soft tissue reconstruction, tissue engineering, translational medicine

## Abstract

Decellularized extracellular matrices (DEMs) have been widely investigated as promising biomaterials for soft tissue augmentation. In contrast, the use of decellularized human amniotic membrane (dHAM) represents a novel approach within this field, offering unique potential for clinical application. The objective of this study is to develop an “off-the-shelf” bioactive soft tissue filler from crosslinked and non-crosslinked dHAM in the injectable microparticle formulation. We used physical and enzymatic processes for decellularization and milling for fabricating the injectable matrix. The decellularization, crosslinking and detoxification protocol removed immunogenic cellular components, preserved collagen and glycosaminoglycan content, and reduced residual crosslinker to sub-cytotoxic levels. Consequently, both crosslinked and non-crosslinked powder extractions promoted cell viability and proliferation and did not affect cell migration in culture. Moreover, the crosslinked powder showed higher stability upon *in vitro* enzymatic degradation and prolonged volume retention in the subcutaneous guinea pig model. Histopathologic evaluation of the implants showed that immune cell infiltration markedly resolved over the 12-week study period. Moreover, immunohistochemistry staining for CD68 and CD163 macrophage markers indicated the abundance of CD163-positive cells, commonly associated with an anti-inflammatory or M2-like phenotype. Staining for CD31 endothelial cell marker demonstrated the formation of blood vessels with intraluminal RBCs and endothelial cell lining within the implants, which is indicative of neovascularization. Therefore, dHAM demonstrated long-term volume retention, reduced immune cell infiltration, and was associated with a CD163-positive macrophage phenotype and evidence of vascularization in the animal model. This study suggests dHAM as a readily deliverable biomaterial and a promising candidate to address clinical translational needs for soft tissue reconstruction.

See also the graphical abstract(Fig. 1).

## List of Abbreviations

DEM: decellularized extracellular matrices

dHAM: decellularized human amniotic membrane

ECM: extracellular matrix

EGFs: epidermal growth factors

FGFs: fibroblast growth factors

GAGs: glycosaminoglycans

H&E: hematoxylin and eosin

HDFs: human dermal fibroblasts

IHC: immunohistochemical

MMPs: matrix metalloproteinases

PDGFs: platelet-derived growth factors

RBCs: red blood cells

SEM: scanning electron microscopy

SLS: static light scattering

VEGFs: vascular endothelial growth factors

## 1. Introduction

In recent years, a variety of biomaterials originating from both synthetic and natural sources have been investigated to address soft tissue volume loss. Among these, extracellular matrix (ECM) bioscaffolds have shown great potential in promoting constructive and functional tissue remodeling across various clinical applications (Cui et al., 2023[8]; Ostadi et al., 2024[25]). ECM-based biomaterials support cell processes such as adhesion, proliferation, and differentiation. Moreover, they are rich in growth factors and signaling molecules that conduct tissue repair and regeneration. However, cellular components of ECM-based materials can be detected by the immune system leading to implant rejection (Kasravi et al., 2023[15]). The decellularization process aims to remove cells and immunogenic components while maintaining the structure of the native ECM and its bioactive elements (Rojas-Murillo et al., 2024[27]). Decellularized ECM (DEM)-based fillers are currently utilized in clinical applications due to their favorable safety profiles and ability to promote in-situ tissue regeneration (Ostadi et al., 2024[25]; Brouki Milan et al., 2025[4]).

Despite extensive research and a robust clinical background, various tissue sources for decellularized biomaterials remain untested as filler materials in clinical settings. Among these, the amniotic membrane stands out as a promising candidate. This homologous, readily available, and cost-effective biological material offers significant potential for scaffolding applications due to its minimal risk of *in vivo* rejection (Buday and Ozturk, 2019[6]; Kafili et al., 2024[13][14]). Although there is a lack of studies specifically exploring decellularized amniotic membranes as soft tissue fillers, evidence from related applications is encouraging. For instance, the ReNu cryopreserved amniotic suspension allograft, a product composed of components of ECM and cells of amniotic fluid, demonstrated greater augmentation in mid-face volume in comparison with platelet-rich plasma (Davis and Augenstein, 2019[9]). Another study investigated a processed product derived from amniotic membrane, incorporated from cellular human amnion, an intermediate layer, and the chorion membrane to be used in soft tissue filling procedures. The scaffold exhibited structural remodeling with new blood vessel formation, and no significant signs of macroscopic inflammation (Moreno et al., 2024[22]). Furthermore, the application of multilayered amniotic membranes implanted into subcutaneous tunnels in rats, as well as amniotic membrane pieces injected subcutaneously, demonstrated long-term volume retention with minimal foreign body reactions, fibrosis, or necrosis (Buday and Ozturk, 2019[6]).

One of the significant challenges in the development of DEM fillers is the biodegradability of these materials, which can deteriorate early clinical outcomes. Degradation of filler materials necessitates costly, frustrating, and invasive replacements. It is shown that crosslinking ECM components improves the biodegradability of DEM fillers and increases resistance to enzymatic degradation (Lee et al., 2015[16]). Moreover, crosslinkers can exert their effects without adversely affecting cell viability (Ahearne and Coyle, 2016[2]). Collagen-based biomaterials are commonly crosslinked using agents such as glutaraldehyde, genipin, carbodiimide, transglutaminase, and reduced riboflavin. Glutaraldehyde, in particular, has been extensively utilized to improve the mechanical strength and durability of ECM-based biomaterials in a variety of clinical applications, including the preservation and crosslinking of bioprosthetic heart valves, patch materials, and decellularized organs (Wassenaar et al., 2016[35]; Yu et al., 2018[37]; Williams et al., 2021[36]; Schwartzman et al., 2023[30]).

This study investigated the development of a bioactive soft tissue filler derived from amniotic tissue. The biomaterial was formulated as an injectable powder micro-particle to enhance the ease of use in a variety of clinical applications. The effect of glutaraldehyde as a crosslinker on the biocompatibility and durability of the filler material was also examined. Decellularized human amniotic membrane (dHAM) powder, in both crosslinked and non-crosslinked forms, was evaluated through *in vitro* analyses and injection into guinea pig subcutaneous tissue. To our knowledge, this study reports the first application of dHAM for soft tissue augmentation.

## 2. Materials and Methods

### 2.1. Preparation of decellularized ECMs

Human amnions were sourced from hospital-provided placentas obtained through planned Cesarean deliveries following uncomplicated pregnancies. Informed consent was obtained from the patients or their guardian(s)/legally authorized representative(s). For the preparation process, the amnion was immediately transferred to the laboratory in a sterile container filled with normal saline solution keeping on dry ice after Cesarean section. Under a laminar flow hood, the amnion membrane was separated from the placenta. This separation involved cutting the amnion/chorion membrane from the placenta and peeling the amnion away from the chorion under sterile conditions. The amnions were then thoroughly rinsed with phosphate-buffered saline (PBS) solution to remove any contaminants from the amnion structure, including small blood clots. Prior to initiating the decellularization process, the amniotic membranes were immersed in an EDTA/trypsin (Merck, Germany) solution for 12 minutes, then scrapped to detach cellular components. To produce the crosslinked extracellular matrix product, the amniotic membranes were immersed in a 0.3 % glutaraldehyde solution for 5 minutes. They were subsequently washed three times with sterile distilled water, each for 5 minutes, to eliminate any residual glutaraldehyde. For the non-crosslinked extracellular matrix product, this step was skipped. The cleaned amniotic membrane was cut into 5 × 5 cm square pieces and placed on pre-sterilized Teflon sheets to air-dry. After 24 hours, the amnion membrane sheets in both crosslinked and non-crosslinked groups were fully air-dried and then mined with surgical scissors and milled into powder by cryomiller (Amin Asia Fanavar Pars, Iran) under a determined protocol. The powdered product was strained through a 325-mesh to eliminate large particles.

### 2.2. Powder characterization

#### 2.2.1. Scanning electron microscopy (SEM) and static light scattering (SLS)

The ultra-structure of the dHAM powder particles was analyzed using scanning electron microscopy (SEM). dHAM powder coated with gold via sputtering for 90 seconds. Imaging was performed using a scanning electron microscope (TESCAN VEGA, Czech Republic). The particle size of dHAM powder in the suspension phase was measured using the static light scattering (SLS) method. The powder was mixed into distilled water, and an automatic particle sizer (FRITSCH, Germany) was used to measure particle size by irradiating a laser beam of 693 nm wavelength. The laser scattering patterns were analyzed to calculate particle size distribution.

#### 2.2.2. DNA quantification

DNA quantification was carried out using a DNA Purification Kit (CinnaGen, Iran) following the manufacturer's protocols. To ensure complete digestion, dHAM was treated with proteinase K at 59 °C for 3 hours. The DNA content was determined by measuring absorbance at 260/280 nm using a Multimode Detector spectrophotometer (BioTek Synergy HTX, USA).

#### 2.2.3. Collagen content and glycosaminoglycan (GAG) content

To evaluate the collagen content in both crosslinked and non-crosslinked dHAM, 20 mg of dHAM powder from each group was homogenized in 100 μl distilled water. Subsequently, 100 μl of 12M HCl was added to the solution, which was then incubated at 120 °C for 4 hours. After incubation, the solution was dried at 90 °C. Then, according to the manufacturer's instructions, the samples were processed and centrifuged at 12,000 rpm for 15 minutes, and the supernatant was used to determine collagen content using the Hydroxyproline Assay Kit (Kiazist, Iran).

To assess GAG content in both crosslinked and non-crosslinked dHAM, 10 mg of dHAM from each group was homogenized in 400 μl of an enzymatic solution (diluent buffer + papain enzyme) and incubated at 65 °C for 16 hours. Following the manufacturer's instructions, samples were processed and centrifuged, and the supernatant was collected and analyzed for GAG content using the GAG Assay Kit (Kiazist, Iran). The results were compared to cellular human amniotic membrane powder (10 mg), which served as the control group.

#### 2.2.4. Residual glutaraldehyde concentration

High-performance liquid chromatography was used to measure the residual glutaraldehyde concentration. 30 mg of Dinitrophenylhydrazine was dissolved completely in 2 mL of phosphoric acid, followed by the addition of 8 mL of acetonitrile. Standards were prepared using 0.1, 0.3, 0.5, 1, and 3 cc of 10 μg/mL glutaraldehyde standard solution, adjusted to a total volume of 9.5 cc with distilled water. Subsequently, 0.5 cc of DNPH solution was added to each standard, thoroughly mixed, and incubated at 37 °C for 90 minutes. The crosslinked dHAM powder was suspended in PBS at a concentration of 40 mg/mL for 7 days, then centrifuged at 5,000 rpm for 10 minutes. The supernatants were collected and filtered through a polyamide membrane. A mixture comprising 0.8 mL of acetonitrile, 1 mL of the sample solution, 0.1 mL of DNPH solution, and 0.1 mL of deionized water was prepared, thoroughly mixed, and incubated at 37 °C for 90 minutes to ensure complete reaction. Chromatographic analysis was performed under the following conditions (Shimadzu, Japan): reversed-phase C18 column; column temperature 25 °C; mobile phase composition of 75 % acetonitrile and 25 % water; flow rate of 1 mL/min; UV detection wavelength at 360 nm; and injection volume of 10 μl. A standard curve was applied to determine glutaraldehyde concentration in the samples (Li et al., 2024[18]).

#### 2.2.5. In vitro degradation

The degradation of dHAM powder, in both crosslinked and non-crosslinked forms, was assessed using an enzymatic degradation test. 20 mg of crosslinked or non-crosslinked dHAM was immersed in 1 mL 0.01 % collagenase solution at 37 °C. For each enzyme-treated sample, a PBS-treated control was assigned. At time intervals of 12 hours, 1 day, and 3, 6, 9, and 12 days, the suspensions from each group were centrifuged at 5,000 rpm for 10 minutes and the supernatant was removed. This centrifugation protocol was optimized to eliminate particles smaller than 220 nm. Such particles are washed out from the tissue through the lymphatic system, do not contribute to the intended filling effect, and are classified as degraded products in this context (Tiantian et al., 2014[32]; Tang et al., 2024[31]). The deposited powder was lyophilized and the weights were compared between the sample and the assigned control. The initial weight (W_i_ for sample, W_ic_ for control) and the dry sample weight at each time point (W_t_ for sample, W_tc_ for control) were recorded. The degree of degradation was expressed as the percentage of weight loss (wt%) according to the following equation:

W (wt%) = ((*W_i_* - *W_t_*) / *W_i_*) / ((*W_ic_* - *W_tc_*) / *W_ic_*) x 100%

#### 2.2.6. Swelling ratio

20 mg of air-dried dHAM powder, in two forms crosslinked and non-crosslinked, were incubated in 1 mL of distilled water at 37 °C for 1, 4, 8, 12, and 24 hours. After the intended period, filter paper was used to remove excess water, and the weight of hydrated product was measured. The swelling rate was determined using the following equation:

*R* = (*M_w_* - *M_d_*) / *M_d_*

Where M_d_ represents the initial dry mass and M_w_ denotes the wet mass.

### 2.3. Cell culture studies

#### 2.3.1. Effects of dHAM on the viability and proliferation of human dermal fibroblasts (HDFs)

An *in vitro* MTT assay was conducted to evaluate the effect of crosslinked and non-crosslinked dHAM powder extract on the relative viability of human dermal fibroblasts (HDFs) in a culture medium. Test sample extracts of crosslinked and non-crosslinked dHAM at concentrations of 0.5 mg, 1 mg, 2 mg, and 4 mg were prepared by incubating them in DMEM medium (Gibco, USA) at 37 °C for 24 hours. Meanwhile, HDFs were seeded into 96-well plates in three replicates for each proposed group. For the proliferation assay a density of 2,000 cells per well, and for the cytotoxicity assay a density of 3,000 cells per well was applied. The cells were incubated in a complete medium containing DMEM medium (Gibco, USA) supplemented with 10 % fetal bovine serum (FBS, Gibco, USA) and 1 % penicillin-streptomycin (v/v, Sigma¬-Aldrich, USA) at 37 °C under 5 % humidified CO_2_ for 24 hours. After 24 hours of incubation, the sample extracts were filtered through a 22-micron filter. The collected liquid extracts were supplemented with 10 % Fetal Bovine Serum and 1 % penicillin-streptomycin (v/v, Sigma¬-Aldrich, USA), then applied to the sub-confluent monolayer of HDFs. The cells were then treated with the extract mediums at 37 °C for 24, 48, 72, and 96 hours. At the end of the proposed time, the extract mediums were replaced with 20 μl MTT solution (0.5 mg/mL in culture medium, Gibco, USA) + 180 μl complete medium, and cells were incubated at 37 °C for an additional 4 hours. After removing the MTT solution, 200 µL of DMSO was added and mixed thoroughly. The resulting color was quantified by measuring absorbance at 540-630 nm using a microplate reader (BioTek, USA). The treatment groups were compared to blank (DMEM with no cells) and control (DMEM + HDFs).

#### 2.3.2. Cell migration test

HDFs were seeded into 24-well plates at 30,000 cells per well density. A sterile pipette tip (100-1000 µL tip) was used to create scratches across the cell layer. To remove non-adherent cells, the cell layer was washed in PBS 1X. The medium was then replaced with 4 mg/mL extract concentration supplemented with 10 % FBS (Gibco, USA) and 1 % penicillin-streptomycin (v/v, Sigma¬-Aldrich, USA). Digital images were captured after 0, 24, and 48 hours, and the remaining scratch area was determined by ImageJ software (NIH, USA). The results were presented as the relative percentage of the remaining scratch area.

### 2.4. Animal studies

#### 2.4.1. Animal study design

The experiments were conducted in accordance with the National Institutes of Health (NIH) guidelines for the care and use of laboratory animals (NIH Publication No. 8023, Revised 1978) and reported in line with the ARRIVE guidelines 2.0. The animal experiment protocols were approved by the Ethics Committee of Shahid Beheshti University of Medical Sciences (Ethical Code: IR.SBMU.AEC.1403.025). Eighteen female wild-type Dunkin Hartley guinea pigs aged 6-8 weeks weighing 400-500 g purchased from Pasteur Institute (Karaj, Iran). Animals were kept with free access to food and drinking water, under the controlled condition of standard temperature (25 ± 1°C) and humidity and grouped 3 per cage with a normal day-night cycle (12:12 h light/dark).

Animals were randomly divided into two groups (using a random number generator) and received either crosslinked or non-crosslinked dHAM powder (9 animals in each group). This sample size was determined based on pilot phase studies to ensure adequate statistical power while minimizing animal use. The guinea pigs were anesthetized with a combination of ketamine 10 % (40 mg/kg, Alfasan, Netherlands) and xylazine 2 % (5mg/kg, Alfasan, Netherlands) solution. dHAM powders were mixed with physiological saline at a concentration of 0.1 g/mL and injected subcutaneously on both the right and left sides of the dorsal region. A control group receiving physiological saline was not included, consistent with previous studies demonstrating that saline administration does not induce long-term volumetric or histopathological changes. 

During the study period, volumetric assessment of implants was performed. Moreover, at each study timepoints of 1, 4, and 12 weeks (n = 3 animals per group per timepoint), the guinea pigs were anesthetized with ketamine/xylazine combination and implants were removed for histological analysis. Then, they were humanely euthanized with CO_2_. All outcome assessments were conducted by investigators blinded to group allocation.

#### 2.4.2. Volumetric assessment

To evaluate the durability of the injected implants, animals were anesthetized, and their implants' volume was measured thorough the skin using a vernier caliper immediately after injection, one day and one week after injection, then every two weeks until the study endpoint. Moreover, the weight of implants was quantified following sacrificing animals at 1, 4 and 12 weeks.

#### 2.4.3. Histological assessment

The harvested implants were fixed overnight in 4 % formalin, followed by paraffin embedding and sectioning into 4-μm thick slices. The sections were then stained with hematoxylin and eosin (H&E) and Masson's trichrome. H&E staining was used to evaluate cell infiltration and angiogenesis, and Masson's trichrome staining was applied to examine alterations in collagen content. To quantify infiltrated cells, images were captured using a microscope (Nikon, Japan) and a digital camera (Nikon, Japan). For each implant, two regions were analyzed separately: the edge (defined as the area within 100 μm of the implant-host interface) and the center (the deep interior of the implant). In each region, 10 randomly selected high‑power fields (HPF; 400× magnification) were captured, and the number of cell nuclei was counted manually. The mean number of cells per HPF was calculated for the edge and for the center of each implant. The edge/center ratio was then derived by dividing the mean edge cell count by the mean center cell count for the same implant. All counts were performed by investigators blinded to group allocation.

#### 2.4.4. Immunohistochemical assessment

To examine infiltrating macrophages and angiogenesis, immunohistochemical (IHC) staining was performed using macrophage markers (CD163 and CD68) and the endothelial cell marker CD31. Harvested implants were fixed overnight in 4 % formalin, embedded in paraffin, and sectioned into 3-μm-thick slices. The sections were blocked at room temperature for 15 minutes using 100 μl of peroxidase-blocking solution. Primary antibodies were added and incubated overnight at 4 °C as follows: CD31 (1:150, Abcam, USA), CD68 (1:1 dilution, Diagnostic Biosystem, USA), and CD163 (1:1 dilution, Zytomed, Germany). To identify bound primary antibodies, the Cell Marque System Detection kit (Sigma-Aldrich, USA) was used, and 100 μl of peroxidase-linked polymer was added to each section and incubated for 30 minutes at room temperature. Diaminobenzidine (DAB) substrate solution (100 μl) was applied to the sections and incubated at room temperature for 10 minutes, followed by counterstaining with hematoxylin for 2 minutes and washing in tap water. Stained sections were observed by microscope (Nikon, Japan) and photographed by digital camera (Nikon, Japan).

### 2.5. Statistical analysis

Pairwise comparisons between two groups were conducted using the independent samples T-test (or dependent samples T-test where the difference in one group across time points was intended). The difference across multiple groups was determined using One-way analysis of variance (ANOVA) with LSD or Tukey's multiple comparisons (where applicable). All statistical analyses were performed in IBM SPSS Statistics (Version 27) and GraphPad Prism Software (Version 10). Visualizations were conducted using GraphPad Prism Software (Version 10). The following analyses were conducted using the Python programming language (version 3.8; www.python.org). To assess differences in particle size distributions between crosslinked and non-crosslinked powders, the Wilcoxon signed-rank test was performed on paired percentile values (D10 to D99.9) using the SciPy library. Additionally, a linear regression model for residual glutaraldehyde quantification was developed using Scikit-learn and visualized with Seaborn. Results were presented as mean ± standard deviation (SD), and P values < 0.05 were considered statistically significant. No datapoint was excluded during analysis.

## 3. Results

### 3.1. Preparation and characterization of injectable dHAM powder

#### 3.1.1. Characterization of particle size and shape

**Figure 2:**
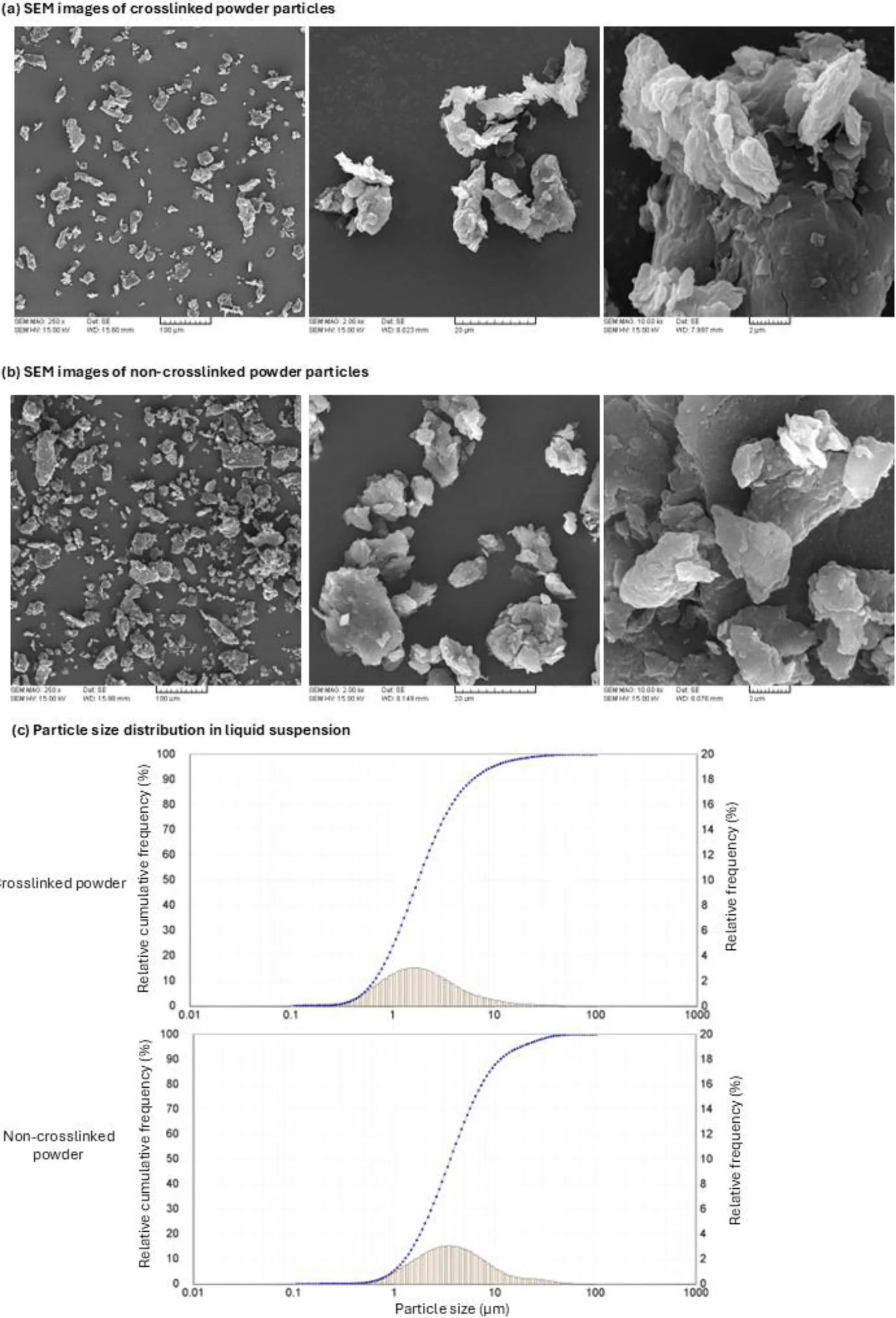
dHAM powder characterization: SEM images of dHAM particles in (a) crosslinked, and (b) non-crosslinked groups. (c) Evaluation of the particle size distribution in the suspension of dHAM powder in distilled water using static light scattering (SLS) technique. The X-axis represents particle size on a logarithmic scale (0.01-1000 μm). Bars indicate the relative frequency of each size bin, while blue dots represent the relative cumulative frequency.

Powder particles were produced by milling air-dried decellularized tissue under reduced temperature and straining by mesh sieve, generating a fine powder. SEM and SLS were used to evaluate particle size and morphology. SEM showed the powder particles are irregular in shape and heterogeneous in size (Figure 2a & 2b(Fig. 2)). Localized particle clustering was observed in certain areas, possibly indicating an inherent tendency for aggregation. The SLS technique evaluated the particle size distribution in suspension in distilled water (Figure 2c(Fig. 2)). The median size of particles was 1.78 μm for crosslinked and 3.62 μm for non-crosslinked powder. The Wilcoxon signed-rank test applied to paired particle size percentiles (D10-D99.9) indicated a statistically significant difference in particle size distribution (W = 0.0, p-value = 0.002), with larger particle sizes observed in the non-crosslinked group. This difference could be attributed to increased brittleness and reduced plasticity induced by crosslinking, which facilitates fragmentation during the milling process and results in finer particles.

The particle size in crosslinked tissue was below 50.82 μm, and in non-crosslinked tissue was below 53.86 μm, accounting for 99.90 % of the particles. This finding indicates that straining powder with steel mesh restricted particle size to those that can effectively pass through 27-gauge needle, which is a standard in aesthetic procedures. No instances of needle occlusion were observed, increasing the reliability of injections in clinical settings and ensuring smooth, uninterrupted delivery of the material.

#### 3.1.2. Validation of decellularization

**Figure 3:**
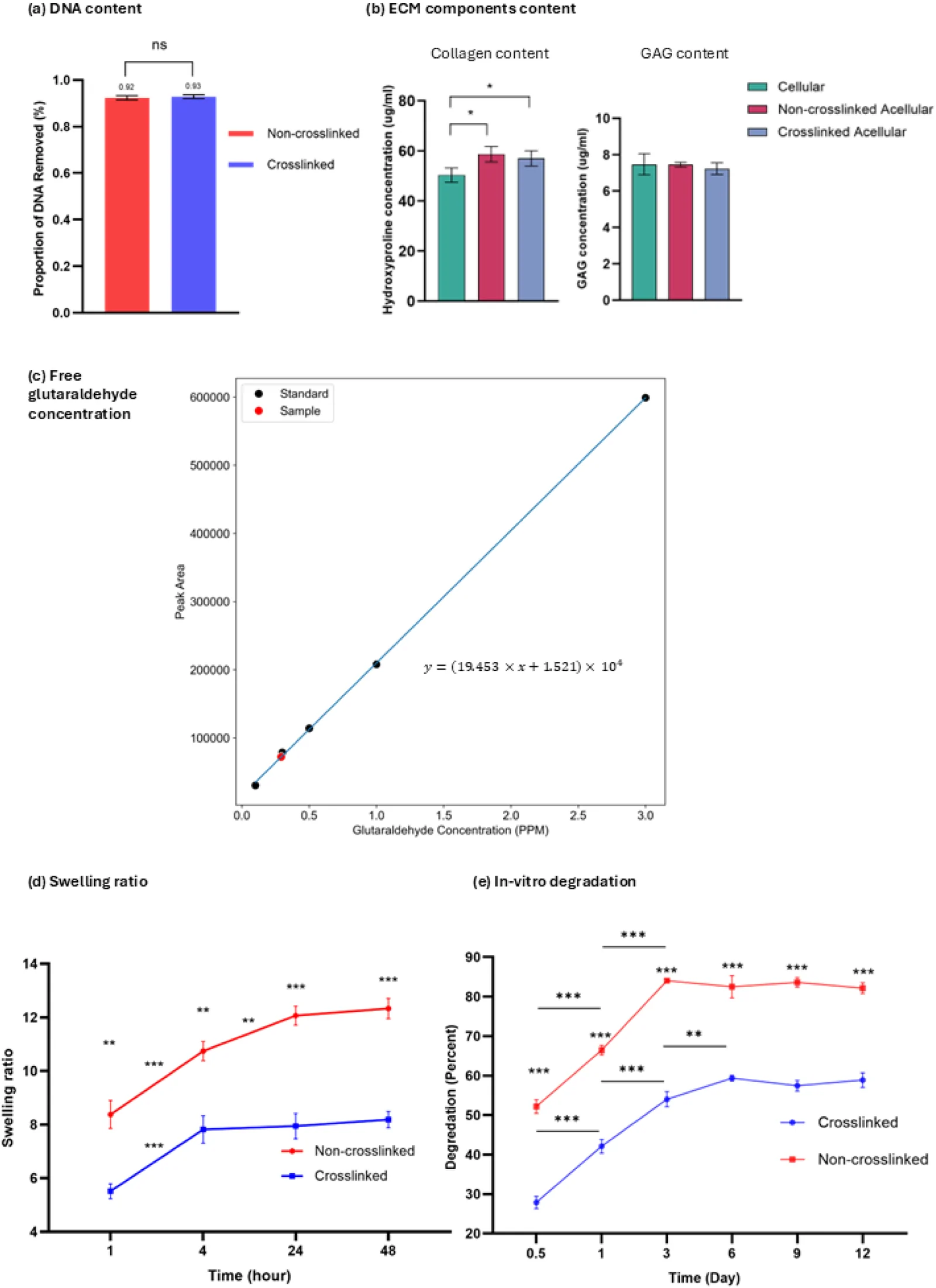
Validation of decellularization and crosslinking processes: The decellularization process effectively removed cellular components while preserving extracellular matrix (ECM) components, and crosslinking enhanced degradation resistance with minimal residual glutaraldehyde. (a) The proportion of DNA removed after the decellularization process in the crosslinked and non-crosslinked groups in comparison with cellular amniotic tissue powder. (b) Collagen and glycosaminoglycan (GAG) content quantification relative to cellular amniotic tissue powder of equal weight. (c) Residual glutaraldehyde levels in 40 mg/mL of crosslinked powder extract were measured by high-performance liquid chromatography (HPLC), with the green dot representing peak area in the sample (attributable to 0.29 μg/mL of glutaraldehyde) and red dots indicating standards (0.1, 0.3, 0.5, 1, and 3 μg/mL of glutaraldehyde). (d) The water uptake capacity of the powders (swelling ratio) and (e) the *in vitro* degradation percent of crosslinked powder (type I collagenase treatment) in comparison with non-crosslinked control. Stars above dots in panel d & e indicate differences between groups, while stars above the lines indicate differences between time points. Error bars represent SD, *P < 0.05, **P < 0.01, ***P < 0.001, ns: non-significant.

The amount of residual DNA was compared in dHAM powder groups and cellular powder control to determine decellularization efficacy. It was shown that more than 92 % of DNA was removed in the decellularization process indicating the successful removal of cellular components from amniotic tissue (Figure 3a(Fig. 3)). Crosslinked and non-crosslinked powders were not different in terms of DNA removal percentage (92.33 ± 0.85 in non-crosslinked vs. 92.82 ± 0.79 in crosslinked group, p-value = 0.94).

#### 3.1.3. Collagen and GAG content

Hydroxyproline assay showed that the concentration of hydroxyproline per mg of powder, as a proxy for collagen content, was higher in the decellularized powders (57.03 ± 3.02 in crosslinked and 58.69 ± 3.10 in non-crosslinked powders) than the native cellular one (50.36 ± 2.83, P < 0.05 for both comparisons, Figure 3b(Fig. 3)). Quantitative assessment of GAGs in tissue powders revealed no significant difference between native cellular and decellularized tissues. These findings indicate that GAGs and collagen are preserved during the decellularization process. Consequently, the removal of cellular components likely resulted in the enrichment of remaining matrix components, particularly collagen.

#### 3.1.4. Free GA measurement

To measure residual GA levels eluted from crosslinked powder samples, the powder was immersed at 40 mg/mL concentration in PBS for 1 week. The residual GA was subsequently quantified using HPLC, and the results showed the mean glutaraldehyde concentration of 0.29 μg/mL for the samples (Figure 3c(Fig. 3)). Considering the two-fold dilution factor applied during GA quantification for samples, and the *in vivo* injection of 100 mg/mL of powder, each milliliter of injected powder suspension would release 1.45 μg of residual GA within a week, less than cytotoxic threshold level determined based on *in vitro* cytotoxicity studies.

### 3.2. Assessment of crosslinking impact through in vitro studies

#### 3.2.1. Swelling ratio

The water uptake capacity of the powders was evaluated by comparing the weight of swollen and air-dried materials over time. Both powders significantly gained weight through absorbing water during the first four hours, after which the swelling ratio plateaued with no significant changes (Figure 3d(Fig. 3)). Throughout all assessed time points, the non-crosslinked powder demonstrated a higher swelling ratio than the crosslinked powder (P < 0.01 at 1 and 4 hours, and P < 0.001 in 24 and 48 hours). At the end of the 48-hour period, the swelling ratio measured 12.30 (95 % CI: 11.95-12.65) for non-crosslinked powder and 8.18 (95 % CI: 7.90-8.46) for crosslinked powder. These findings suggest that non-crosslinked powders possess a greater water uptake capacity, potentially influencing their hydration behavior.

#### 3.2.2. In vitro degradation

The *in vitro* degradation of powders was evaluated following treatment with type I collagenase. The degradation rate in the non-crosslinked powder increased up to day 3 before reaching a plateau, while the crosslinked powder exhibited a plateau at day 6 (Figure 3e(Fig. 3)). In all the assessed time points, the degradation rate was higher in the non-crosslinked group than in the crosslinked group (P < 0.001 at all time points). By the end of the 12-day period, the degradation rate was 82.17 ± 1.36 % in the non-crosslinked and 58.87 ± 1.86 % in the crosslinked group. These findings confirm that crosslinking significantly prolongs the enzymatic stability of the dHAM biomaterial.

### 3.3. Cell culture

#### 3.3.1. In vitro cytotoxicity of dHAM powder on HDFs

**Figure 4:**
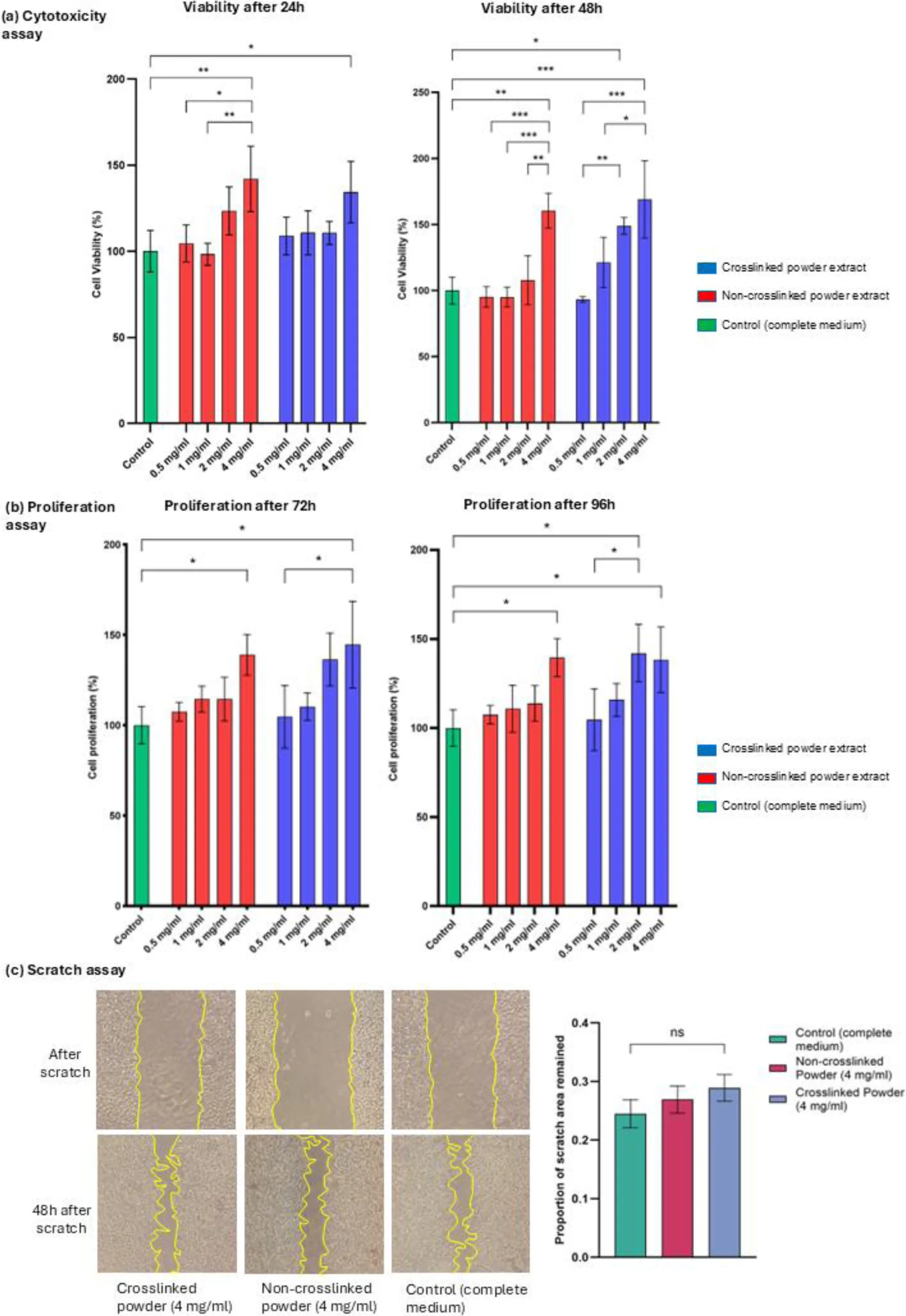
Effect of hDAM powder extracts on HDF culture: Both crosslinked and non-crosslinked dHAM powder extracts supported human dermal fibroblast (HDF) viability and proliferation without influencing cell migration. (a) *In vitro* cytotoxicity analysis of the effect of various concentrations (0.5 mg/mL to 4 mg/mL) of dHAM extract on HDFs after 24 hours and 48 hours of cell treatment. (b) Proliferation test results of the effect of dHAM extract in various concentrations (0.5 mg/mL to 4 mg/mL) on HDFs after 72 hours and 96 hours of cell treatment. Note: Values in viability and proliferation tests are expressed as a percentage relative to the control group (untreated cells), which was set to 100 %. Therefore, values above 100 % indicate higher metabolic activity than the control group. (c) Scratch area remained in wells treated with crosslinked and non-crosslinked powder extracts (4 mg/mL), in comparison with complete medium control. Representative microscopic images in 4× magnification are provided. Error bars represent SD, *P < 0.05, **P < 0.01, ***P < 0.001, ns: non-significant.

MTT assay was conducted to determine the effect of dHAM extraction at various concentrations (0.5 mg/mL to 4 mg/mL) on HDFs. After 24 hours of incubating cells with extracts, both crosslinked and non-crosslinked powder extracts at 4 mg/mL concentration showed higher cell viability than complete medium control (P < 0.05 for crosslinked and P < 0.01 for non-crosslinked groups) (Figure 4a(Fig. 4)). After 48 hours, crosslinked powder extract at two extract concentrations of 4 mg/mL (P < 0.001) and 2 mg/mL (P < 0.05), as well as non-crosslinked extract at 4 mg/mL concentration (P < 0.01) demonstrated increased cell viability relative to the control (Figure 4a(Fig. 4)). Overall, higher concentrations of powder extracts were associated with enhanced cell viability.

#### 3.3.2. Proliferation of HDFs under dHAM extract

To determine the effect of dHAM extracts on HDF proliferation, the duration of incubating HDFs with extracts was extended to 96 hours. At 72 hours incubation period, both crosslinked and non-crosslinked powder extracts at the concentrations of 4 mg/mL (P < 0.05) showed stimulatory effect on cell proliferation relative to complete medium control (Figure 4b(Fig. 4)). After 96 hours, crosslinked powder extract at two concentrations of 4 mg/mL and 2 mg/mL (P < 0.05 for both concentrations), and non-crosslinked powder extract at 4 mg/mL concentration (P < 0.05), showed stimulatory effect on cell proliferation. Therefore, dHAM powder extracts promoted HDF proliferation, with higher extract concentrations showing a greater stimulatory effect.

#### 3.3.3. Migration of HDFs under dHAM extract

A scratch wound assay was performed to investigate the effect of dHAM extract (4 mg/mL) on HDF migration. Cell migration was monitored at 0, 24, and 48 hours post-incubation, and the percentage of remaining wound area at 48 hours was quantified (Figure 4c(Fig. 4)). There was no significant difference in the remaining wound area between crosslinked extract (28.90 ± 2.27), non-crosslinked extract (26.93 ± 2.31), and complete medium control (24.50 ± 2.41). Therefore, we did not capture any stimulatory or inhibitory effects of dHAM on cell migration.

### 3.4. In vivo injection of dHAM

#### 3.4.1. Volume retention

**Figure 5:**
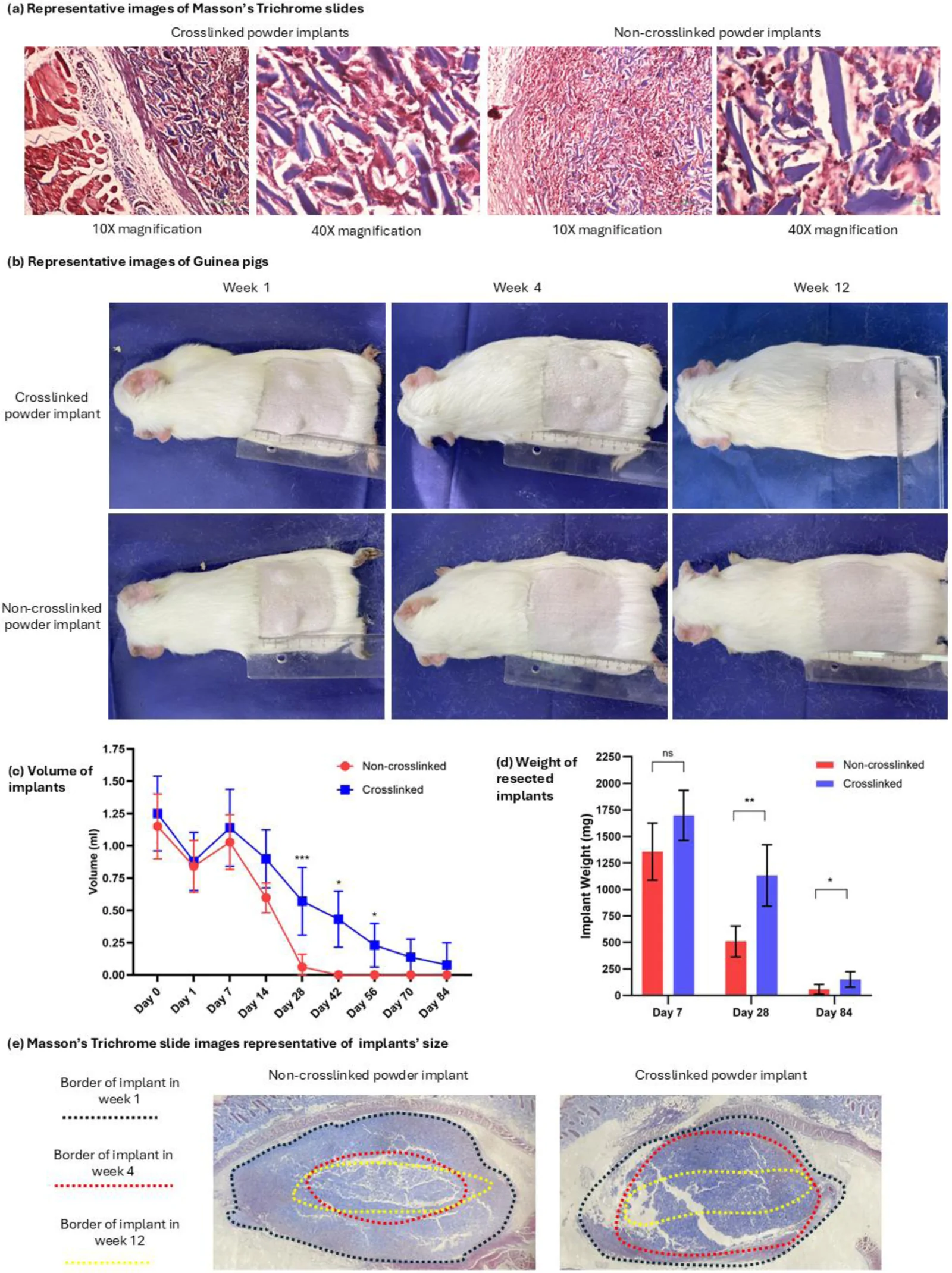
Preservation of ECM components and volume of injected implant in the guinea pig model: Staining for collagen fibers demonstrated that the implants were able to preserve their collagen throughout the study period. Moreover, crosslinked implants demonstrated greater volume retention in the animal model. (a) Masson's Trichrome staining of implants in week 4, in which collagen fibers are represented in blue color and cellular infiltrations are stained red. (b) Representative images of guinea pigs, demonstrating noticeable differences in lump size between groups. (c) The graph of implant's volume from post-injection (day 0) to the end of the study. (d) The graph of implants' weight at excision timepoints (n = 3). (e) Masson's Trichrome staining of implants excised after 1, 4, and 12 weeks, with implant boundaries shown by dashed lines. The stars above the dots in panel B indicate the difference between groups on the specified time point. Error bars represent SD, *P < 0.05, **P < 0.01, ***P < 0.001, ns: non-significant.

We evaluated and compared the volume retention of crosslinked and non-crosslinked dHAM powder injection in the guinea pig model. Guinea pigs (n = 9 per group) received powder suspension (100 mg/mL) subcutaneously in the back region. Masson's Trichrome staining showed that implants preserved their collagen composition four weeks after subcutaneous injection (Figure 5a(Fig. 5)). Volumetric assessment showed a gradual reduction in the implant volumes after the first week and enhanced volume retention in the crosslinked implants in comparison with non-crosslinked ones (Figure 5b & 5c(Fig. 5)). By week 6, none of the non-crosslinked implants exhibited measurable volume, although they remained palpable. In contrast, most crosslinked implants retained measurable volume up to week 10. The crosslinked powder implants had a significantly higher volume than non-crosslinked implants on week 4 (0.57 vs 0.06 cc, P < 0.01), week 6 (0.43 vs. 0.00 cc, P < 0.05), and week 8 (0.23 vs. 0.00 cc, P < 0.05). The implants were excised in weeks 1, 4, and 12, and their weights were recorded (Figure 5d(Fig. 5)). The weight in the crosslinked group was higher than the non-crosslinked group in week 4 (1.13 vs. 0.51 gr, P < 0.01) and week 12 (0.15 vs. 0.06 gr, P < 0.05). Histopathological slides of the recovered implants also confirmed the gradual volume loss from week 1 to week 12 and marked difference in implant boundaries in week 4 (Figure 5e(Fig. 5)). These findings indicate that crosslinking effectively prolongs implant's volume retention, suggesting potential clinical benefits for achieving a more durable filling effect.

#### 3.4.2. Immune response

**Figure 6:**
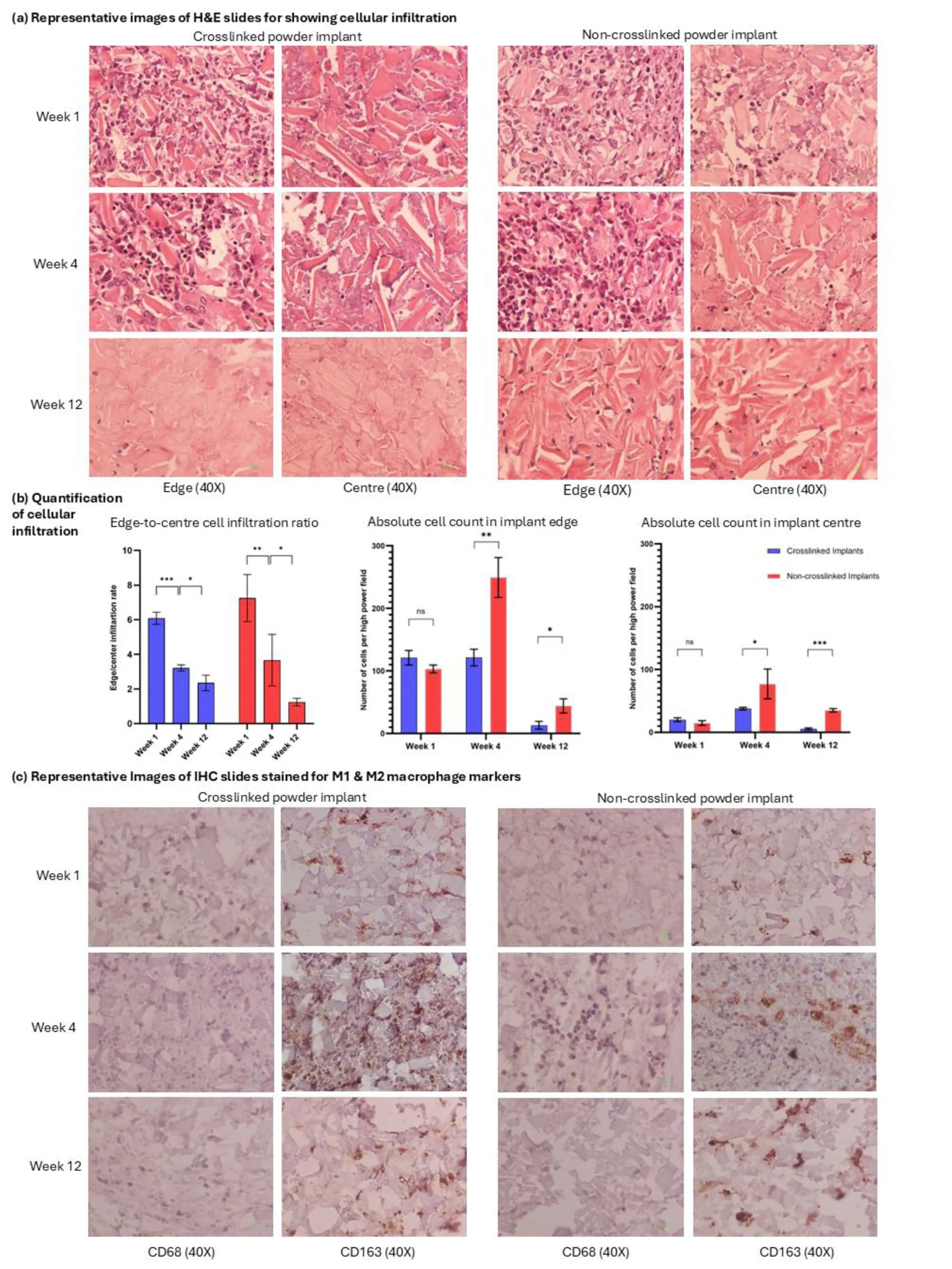
Immune response to dHAM implants: Implants were able to subside cellular infiltration over 12 weeks and maintain a macrophage population exhibiting a CD163-positive (M2-like) phenotype. (a) Images of the edge and center of dHAM implants in high magnification showing cellular infiltration. (b) Edge-to-center cell infiltration ratio and absolute cell counts in these regions in crosslinked and non-crosslinked implants. (c) high magnification images of slides stained for CD68 and CD163 markers. Error bars represent SD, *P < 0.05, **P < 0.01, ***P < 0.001, ns: non-significant.

Cellular infiltration into the implants was quantified to evaluate the immune response to dHAM implants. A general paradigm for cellular infiltration of the implants was the predominance of infiltration in the edge of the implants rather than the center (Figure 6a & 6b(Fig. 6)). The rate of edge-to-center cellular infiltration reduced from 6.05 ± 0.38 in week 1 to 2.38 ± 0.47 in week 12 in crosslinked implants. Similarly, the rate decreased from 7.27 ± 1.38 in week 1 to 1.24 ± 0.22 in week 12 in non-crosslinked implants (Figure 6b(Fig. 6)). Therefore, cellular infiltration became more homogenous over time. Moreover, the number of infiltrated cells markedly reduced in 12-week implants, which indicates the resolution of inflammation by this week (Figure 6a & 6b(Fig. 6)). In both crosslinked and non-crosslinked implants in both the edge and center of implants, cellular infiltration peaked at week 4 and resolved through week 12 (Figure 6a & 6b(Fig. 6)). Interestingly, crosslinked implants had lower cellular infiltration than non-crosslinked implants in week 4 (P < 0.01 for edge; P < 0.05 for center) and week 12 (P < 0.05 for edge; P < 0.001 for center). Therefore, bio-scaffold implants were able to resolve cellular infiltration over the 12-week study period, and crosslinked implants were superior in this regard.

Considering that macrophages are key contributors to the immune response to DEMs, we assessed macrophage phenotypes using immunohistochemistry. CD68 was used as a general macrophage marker, while CD163 was used as a marker commonly associated with an anti-inflammatory or M2-like phenotype. Staining for CD68 demonstrated that CD68-positive cells were scarce in both crosslinked and non-crosslinked implants across all study time points (Figure 6c(Fig. 6)). In contrast, CD163-positive cells were observed abundantly in all crosslinked and non-crosslinked implants at all time points. These findings suggest a predominance of CD163-positive macrophages, which is consistent with an M2-like bias.

#### 3.4.3. Angiogenesis

**Figure 7:**
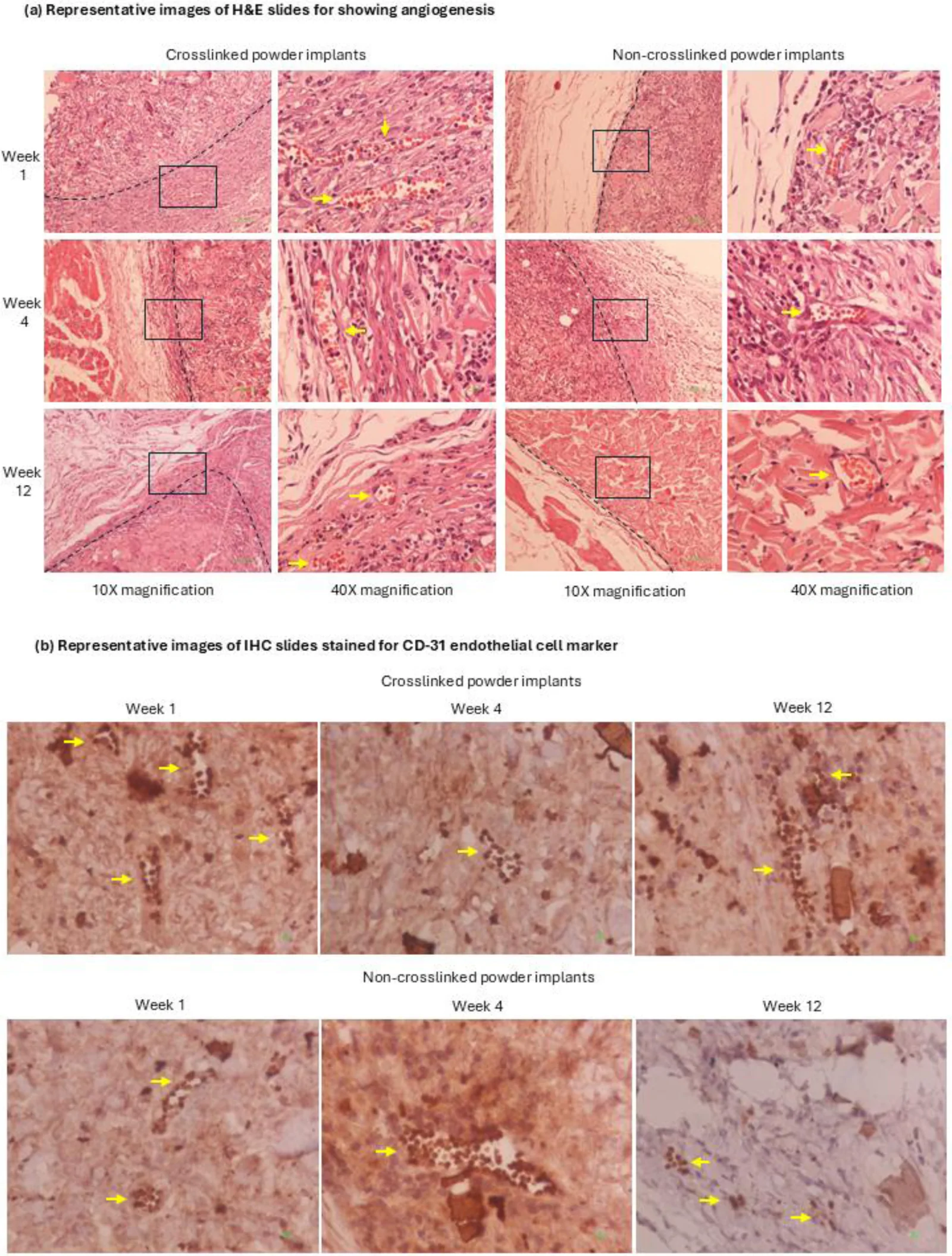
Angiogenesis: The implants showed evidence of angiogenesis at all study time points. (a) Representative H&E-stained images of implants at 10× magnification, with 40× magnification insets highlighting vascular structures and inner RBCs. (b) High-magnification (40×) images of CD31 IHC-stained slides, showing endothelial cell-lined blood vessels. Dashed lines indicate the implant border, and yellow marks indicate blood vessels.

After the implantation of DEM scaffolds in host tissue, the formation of new blood vessels is essential for supporting cellular infiltration from host tissue and integration of implants into body. In this study, H & E and CD31 IHC staining were utilized to demonstrate the formation of blood vessels. H & E-stained images demonstrated the presence of blood vessels characterized by intraluminal red blood cells (RBCs) within the implant, the surrounding capsule, and the peri-implant region (Figure 7a(Fig. 7)). Similarly, CD31 IHC confirmed the formation of endothelial cell-lined vessels containing RBCs within the implant, which is supporting evidence of neovascularization (Figure 7b(Fig. 7)). These findings were consistent across both crosslinked and non-crosslinked implants at all study time points. Hence, the implants exhibited evidence of vascularization, which is necessary for transferring blood and nutrients to the scaffold.

## 4. Discussion

ECM is a three-dimensional network composed of collagen, elastin, glycosaminoglycans, fibronectin, laminin, glycoproteins, and proteoglycan (Cui et al., 2023[8]). The remaining physicochemical signals and biological performance in DEMs would be a substrate for native ECM (Zhang et al., 2021[39]). Superior bioactivity is the main advantage of DEMs over conventional fillers, which is due to retaining ECM components required for tissue regeneration (Ostadi et al., 2024[25]). Therefore, ongoing research is being conducted to explore the potential of DEM fillers to replace conventional filler materials. Decellularized biomaterials derived from dermal and adipose tissues were promising in this regard. Acellular dermal matrices (ADMs) are obtained from various biological sources, including human, porcine, and bovine tissues. Acellular adipose matrices (AAMs) are derived from the abundant adipose tissue typically discarded during procedures such as lipectomy, abdominoplasty, or breast reduction. It is shown that these biomaterials retain physiochemical cues to support stem cell proliferation, angiogenesis, and recellularization as filler materials; however, the risk of immunogenicity and lack of long-term stability has limited their application (Mohiuddin et al., 2020[21]; Ostadi et al., 2024[25]).

dHAM has extensive research backgrounds in many clinical settings, while it is less studied for soft tissue augmentation (Kafili et al., 2024[13][14]; Ostadi et al., 2024[25]). As mentioned, a challenge in the application of DEM filler materials is their rapid biodegradation, which shrinks the injected or implanted filler volume. Matrix metalloproteinases (MMPs) are one of the major contributors to DEM filler degradation. Previous studies have shown that MMP enzymes such as MMP-2, MMP-8, and MMP-9 are highly outnumbered by inhibitors of MMPs such as TIMP-1, TIMP-2, α2M, and A1AT in the amniotic/chorionic membrane. Moreover, it is shown that MMPs are mainly in their latent form or complex with inhibitors in this tissue (Lei et al., 2017[17]). The finding recommends that decellularized amniotic tissue may remain largely unaffected by MMP activity, making it a promising option for long-lasting soft tissue augmentation. On the other hand, amnion is a readily accessible and low-cost tissue source with remarkable biological properties such as biocompatibility, low immunogenicity, anti-scarring, anti-inflammatory, and antibacterial effects (Kafili et al., 2024[13]). The dHAM has been demonstrated to preserve ECM components and growth factors. Consequently, dHAM not only provides a supporting scaffold with lower immunogenicity but also promotes cell proliferation, differentiation, and adhesion (Doudi et al., 2022[10]). According to the conspicuous features, the potential application of amniotic membrane as a filler biomaterial was investigated in this study.

In this study, the decellularization protocol was composed of the enzymatic treatment of amniotic membranes with EDTA/trypsin enzyme followed by scraping to loosen epithelial cells. After air-drying decellularized amniotic membrane sheets, dHAM powder was prepared by milling the sheets at reduced temperatures, followed by straining through a mesh sieve. The evidence for the removal of cellular remnants was shown by more than 92 % reduction in DNA content of decellularized powder in comparison with cellular one. Moreover, the collagen and GAG content remained unchanged. These data indicate that the decellularization protocol effectively preserved ECM structures while eliminating immunogenic cellular components. Collagen and GAG macromolecule families are the predominant compositional fractions of ECM and derive the most widely used filler materials consisting of collagen and hyaluronic acid fillers. Therefore, the preservation of these two components can essentially address tissue bulking in the injected site (Aamodt and Grainger, 2016[1]).

Crosslinked and non-crosslinked powders were also compared in terms of biodegradability and cytotoxicity in the *in vitro* settings, and volume retention and biocompatibility in animal models. The dHAM powder in both crosslinked and non-crosslinked forms demonstrated a favorable safety profile *in vitro*. The powder extraction not only showed no *in vitro* cytotoxicity on human dermal fibroblasts but also supported cell viability and proliferation in higher extract doses. However, due to the nature of the MTT assay, the higher optical density observed at 24 h and 48 h (viability) and at 72 h and 96 h (proliferation) could be attributed either to an increased number of cells or to enhanced cellular metabolic activity. The potential for increase in cell number is supported by previous studies showing that decellularized matrices can enhance cell proliferation by approximately 1.5-fold (Cui et al., 2023[8]). The supporting effect could be due to preserved growth factors in the structure of dHAM, including epidermal growth factor (EGF), vascular endothelial growth factors (VEGFs), fibroblast growth factors (FGFs), and platelet-derived growth factors (PDGFs) (Lei et al., 2017[17]). These growth factors are well recognized for their regenerative and pro‑proliferative roles in wound healing and tissue engineering (Lei et al., 2017[17]). In parallel, the increased MTT reduction may also reflect direct stimulation of mitochondrial function by the dHAM matrix. Indeed, accumulating evidence indicates that extracellular matrix signals can promote mitochondrial activity through enhancing mitochondrial respiration and membrane potential (Zhang et al., 2023[40]; Gatti et al., 2026[11]). Further studies are needed to determine the relative contribution of proliferation versus enhanced mitochondrial activity to the observed increase in MTT reduction.

The crosslinked powder showed lower *in vitro* biodegradation when treated with collagenase enzyme and better *in vivo* volume retention. This higher volume retention is particularly crucial in clinical applications, where rapid biomaterial degradation can lead to injected volume shrinkage, suboptimal outcomes, and the need for costly, frustrating, and invasive filler replacements. The higher implant volume in the crosslinked group was in line with previous studies, demonstrating that cross-linkers can improve the resistance of DEMs to enzymatic degradation without detrimentally affecting cells' viability or inducing destructive immune responses (Ahearne and Coyle, 2016[2]). Moreover, we have shown fewer cellular infiltration in crosslinked implants than in non-crosslinked ones, which is in accordance with some previous studies that indicate cross-linking can modulate immune response (Umashankar et al., 2013[33]).

Interestingly, the clinically significant increase in volume retention was achieved by treating the amniotic membrane with low concentrations of glutaraldehyde (0.3 %) in a limited time span (5 min). Moreover, detoxification of membranes from residual glutaraldehyde was achieved by washing them in distilled water in three 5-minute intervals. HPLC confirmed the release of 1.45 μg of residual glutaraldehyde per 100 mg of crosslinked powder after one week of suspension in PBS. Considering that each milliliter of the injected filler would release 1.45 μg residual glutaraldehyde within a week, and the glutaraldehyde would be rapidly diluted in the body, our data suggest the amount of residual glutaraldehyde would be by far less than 3 μg/mL cytotoxic threshold level determined based on *in vitro* cytotoxicity studies (Casali et al., 2018[7]). Our approach of quantifying residual GA release and comparing it to an *in vitro* cytotoxicity threshold is consistent with previous studies evaluating safety of glutaraldehyde crosslinked biomaterials (Park et al., 2020[26]; Li et al., 2024[18]). Accordingly, the present data can be considered a valid initial safety assessment for the crosslinked dHAM filler, supported by in‑vitro cytotoxicity and animal studies. However, we acknowledge that factors such as local concentration at the implant site, release kinetics (potential bolus effect versus gradual elution), and the body's capacity for metabolism and clearance are not fully captured by this *in vitro* analysis. Therefore, a comprehensive toxicological risk assessment would be required to definitively confirm clinical safety.

The crosslinked and non-crosslinked powder implants showed favorable immune response outcomes in the *in vivo* setting. DEM biomaterials may elicit an immune response that can be constructive or destructive for the implant, depending on the extent of immune system activation and allorecognition (Ostadi et al., 2024[25]). Both innate and adaptive immune cells contribute to this response, and their phenotype can determine downstream repair and biocompatibility outcomes (Anderson et al., 2022[3]). A constructive immune response to DEM scaffolds is characterized by transient innate immune activation and the dominance of the anti-inflammatory M2 macrophage phenotype over the pro-inflammatory M1 phenotype (Anderson et al., 2022[3]; Ostadi et al., 2024[25]). It is shown that in the constructive immune response, implanted DEM biomaterials can effectively induce macrophage polarization from M1 to M2 within 7 to 14 days (Brown et al., 2014[5]; Lin et al., 2019[19]). In the present study, apart from quantifying infiltrated immune cells into implants, we assessed CD68 and CD163 expression using immunohistochemistry. CD68 is widely considered a pan-macrophage marker, whereas CD163 is commonly associated with an anti-inflammatory or M2-like phenotype. This approach has been useful in predicting *in vivo* ECM scaffold implantation outcomes (Anderson et al., 2022[3]; Cui et al., 2023[8]; Walker et al., 2025[34]). We observed that the injected implants were abundantly populated with CD163-positive macrophages as early as the first week. Unexpectedly, CD68-positive cells were scarce. One possible explanation is that CD68 is highly expressed on pro-inflammatory M1 macrophages but is present at lower levels on M2-like cells (Scala et al., 2021[29]); therefore, the low number of CD68-positive cells may reflect a relative scarcity of M1-like macrophages rather than an absence of macrophages overall. Moreover, we showed that the infiltrated immune cell population subsided in the period between weeks 4 and 12 after implantation. These results suggest that the biomaterial initiated the resolution phase of inflammation and promoted a microenvironment conducive to constructive tissue remodelling. Nevertheless, it is important to acknowledge that CD163 alone does not definitively establish M2 polarization, and additional markers (e.g., iNOS, CD206, Arg-1) would be required for complete characterization.

ECMs served as potent angiogenic materials since they provide factors essential for driving microvessel formation, including VEGFs, FGFs, PDGFs, and angiopoietins (Zhang et al., 2021[38]; Ostadi et al., 2024[25]; Melnick et al., 2025[20]). Moreover, current evidence establishes that the immune system response to DEMs, especially implant-resident macrophages, has an indispensable role in angiogenesis (Nolfi et al., 2020[24]). H&E-stained slides showed the formation of blood vessels with RBCs in their lumen in all the 1-, 4-, and 12-week implants. Immunohistochemistry for CD31 endothelial cell marker also demonstrated angiogenesis in all the assessed samples. Therefore, the dHAM implants were able to support angiogenesis, which can drive implant recellularization and integration into host tissue (Cui et al., 2023[8]; Ostadi et al., 2024[25]). Prior research indicates that the presence of M2 macrophages in DEM hydrogel may promote vascularization, while the M1 macrophages inhibited angiogenesis (Cui et al., 2023[8]). Therefore, the CD163-positive macrophages (consistent with an M2-like phenotype) observed in the implant may have a supportive role in the observed angiogenesis, although further studies are needed to establish a direct causal relationship.

DEM scaffolds are used in wide range of clinical applications, such as breast reconstruction, facial filler, and rhinoplasty (Ostadi et al., 2024[25]). The methods used to implant DEMs into host tissues range from surgical interventions to minimally invasive injections. In some applications, such as lip and cheek augmentation, physicians require materials capable of passing through thin needles. Hyaluronic acid fillers, which are widely applied as facial fillers, are typically injected through 27- to 30-gauge needles (Rowland Payne et al., 2023[28]). Using fillers compatible with higher gauge needles ameliorates the injection process by increasing the accuracy of biomaterial delivery and placement, resulting in better symmetry and balance. Moreover, less injection pain, limited bleeding/bruising, and enhanced patient satisfaction are expected (Rowland Payne et al., 2023[28]). Previous studies have reported the injection of DEM fillers using needles with gauge sizes between 18 and 25 (Gold et al., 2020[12]; Nguyen and Tran, 2021[23]). Here, we injected powder suspension into guinea pig subcutaneous tissue through 27-gauge needles, which improves the clinical translatability of the filler product. In this study, the dHAM powder was milled under controlled frequency and time and then strained through a 325-mesh sieve to omit large particles. The milling/straining process resulted in particle sizes of less than 54 μm for the non-crosslinked and 51 μm for the crosslinked powder in the suspension phase, accounting for 99.9 % of the particles. The size range was found to be critical for passing powder particles through 27-gauge needles without occasional occlusions.

## 5. Conclusions

dHAM powder suspension demonstrated a favorable *in vitro* cytotoxicity profile and durable volume retention in the animal model without showing any adverse immune-mediated response. Moreover, crosslinking with low concentrations of glutaraldehyde in a limited time span prolonged DEM implant volume retention, while not showing any cytotoxic effects. The implants were abundantly populated with CD163‑positive macrophages (suggestive of an anti‑inflammatory phenotype) and showed histological evidence of vascularization in the animal model. Therefore, for the first time, this study suggests dHAM powder as a promising candidate for long-lasting soft tissue augmentation and reconstruction.

## Notes

Javad Khanali and Yasamin Ostadi contributed equally as first author.

## Declaration

### Acknowledgments

The authors would like to thank the operation room personnel of Erfan and Eghbal Hospitals, Tehran, Iran for their efforts in obtaining placentas used in this study.

### Conflict of interest

All authors declare no financial or non-financial competing interests.

### Funding

This work was supported by the Vice-Chancellor in Research Affairs, Shahid Beheshti University of Medical Sciences, Tehran, Iran, and the Iran National Science Foundation (INSF), Tehran, Iran [Grant number 40404354]. Authors affirm not having entered into an agreement with the funder that may have limited their ability to complete the research as planned and indicate that they have had full control of all primary data.

### Data availability

All raw data generated and analyzed during this study are included in the supplementary information (Supplementary Data Raw Measurements.xlsx, containing 10 tables with individual data points for all quantitative experiments). Additional datasets are available from the corresponding author upon reasonable request.

### Ethics approval

The protocol of this study including both animal studies and obtaining human placenta samples was approved under the title “Evaluation of the effect of Injecting crosslinked and noncrosslinked human amniotic membrane powder suspension as soft tissue filler in the guinea pig model” by the ethics committee of Shahid Beheshti University of Medical Sciences (Approval number: IR.SBMU.AEC.1403.025) on August 4, 2024.

### Patient consent

The patients or their guardian(s)/legally authorised representative(s) provided written informed consent for the use of the samples (placentas).

### Author contributions

Conceptualization, H.N., J.K., Y.O.; Investigation, J.K., Y.O., F.A.T, T.T., R.T, Y.Y., Y.N., M.S.; Data curation, J.K., Y.O.; Formal analysis, J.K., Y.O.; Writing - Original Draft, J.K., Y.O.; Writing-Review & Editing, H.N., F.A.T, T.T., R.T, Y.Y., Y.N., M.S., F.N., S.B.; Methodology, J.K., Y.O., F.A.T., H.N.; Funding acquisition, H.N.; Resources, H.N.; Project administration, H.N.; Supervision, H.N. All authors have read and approved the manuscript prior to submission.

### Artificial Intelligence (AI) - assisted technology

The authors declare that they have not used AI-generated work in this manuscript.

### Clinical trial registration

Not applicable.

### Permission to reproduce material from other sources

Not applicable.

## Supplementary Material

Supplementary data

## Figures and Tables

**Figure 1:**
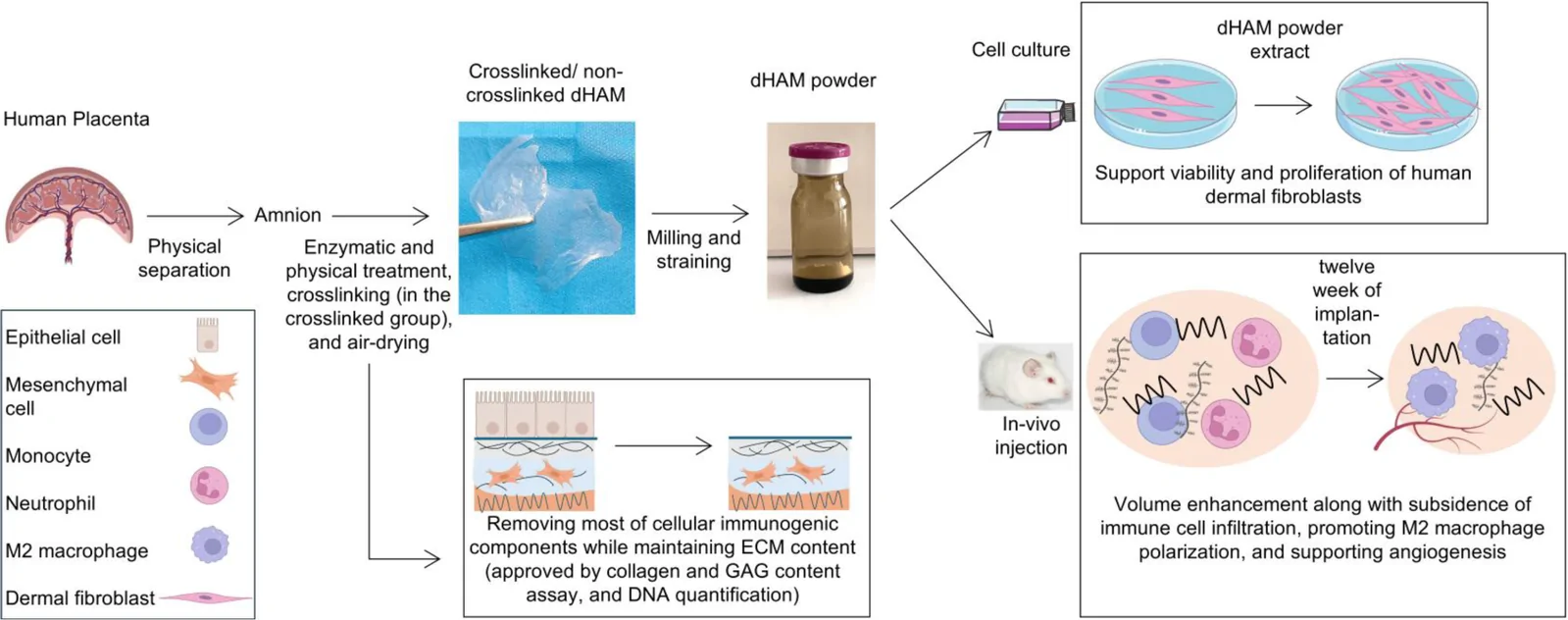
Graphical abstract
